# Generation of a Maize B Centromere Minimal Map Containing the Central Core Domain

**DOI:** 10.1534/g3.115.022889

**Published:** 2015-10-26

**Authors:** Nathanael A. Ellis, Ryan N. Douglas, Caroline E. Jackson, James A. Birchler, R. Kelly Dawe

**Affiliations:** * Department of Plant Biology, University of Georgia, Athens, Georgia 30602; † Division of Biological Sciences, University of Missouri, Columbia, Missouri 65211; ‡ Department of Genetics, University of Georgia, Athens, Georgia 30602

**Keywords:** B chromosome, centromere, maize, misdivision, retroelement

## Abstract

The maize B centromere has been used as a model for centromere epigenetics and as the basis for building artificial chromosomes. However, there are no sequence resources for this important centromere. Here we used transposon display for the centromere-specific retroelement CRM2 to identify a collection of 40 sequence tags that flank CRM2 insertion points on the B chromosome. These were confirmed to lie within the centromere by assaying deletion breakpoints from centromere misdivision derivatives (intracentromere breakages caused by centromere fission). Markers were grouped together on the basis of their association with other markers in the misdivision series and assembled into a pseudocontig containing 10.1 kb of sequence. To identify sequences that interact directly with centromere proteins, we carried out chromatin immunoprecipitation using antibodies to centromeric histone H3 (CENH3), a defining feature of functional centromeric sequences. The CENH3 chromatin immunoprecipitation map was interpreted relative to the known transmission rates of centromere misdivision derivatives to identify a centromere core domain spanning 33 markers. A subset of seven markers was mapped in additional B centromere misdivision derivatives with the use of unique primer pairs. A derivative previously shown to have no canonical centromere sequences (Telo3-3) lacks these core markers. Our results provide a molecular map of the B chromosome centromere and identify key sequences within the map that interact directly with centromeric histone H3.

Centromeres are structural features of the genome that serve as contact points between chromosomes and the spindle during cell division. In maize, the primary centromere sequences are 156-bp tandem repeats called CentC, and a family of retrotransposons called Centromeric Retroelements (CRM elements) (Ananiev *et al.* 1998c; Jiang *et al.* 2003). CentC occurs in continuous arrays extending for hundreds of kilobases, and CRM elements are clustered in nested arrangements with less-common retrotransposons (Jin *et al.* 2004; Wolfgruber *et al.* 2009). Genes and other single copy sequences are rare in centromeres. Nevertheless, transposon junctions and restriction-site polymorphisms made it possible to use standard bacterial artificial chromosome (BAC) sequencing pipelines to assemble maize centromeres 2 and 5 in their entirety (Wolfgruber *et al.* 2009). These sequences have been invaluable references for interpreting centromere structure and evolution (Gent *et al.* 2012; Wang *et al.* 2014; Bilinski *et al.* 2015; Gent *et al.* 2015). However, continued progress on assembling centromeres represents a major challenge, because although BAC assemblies can be powerful tools, they do not traverse all centromeres and are not available for most species.

Even where centromere sequences are known, the functional domains cannot be identified by sequence alone because centromere specification is heavily influenced by epigenetic factors (Allshire and Karpen 2008). In most species, the functional centromere domains are operationally defined by the presence of the centromere-specific histone H3 variant cenH3/CENP-A [centromeric histone H3 (CENH3) in plants], the basal-most centromere protein that serves to recruit overlying kinetochore proteins (Black and Bassett 2008). Chromatin immunoprecipitation with antibodies to CENH3 followed by high-throughput sequencing (ChIP-seq) is considered the gold standard for defining centromere position. Interpreting ChIP-seq requires that there is a reference sequence that has sufficient polymorphism to unequivocally map short sequence reads. Although centromeres generally are composed of tandem repeats and transposons, the fact that they are interspersed with each other in maize (and other plants) provides for a remarkable degree of polymorphism (Luce *et al.* 2006; Shi *et al.* 2010). The maize CRM family contains many variants and over millions of years have inserted into each other and into other repeats in random fashion (Sharma and Presting 2008). This diversity has been exploited by the use of a technique called transposon display, whereby primers to the long terminal repeat (LTR) of a common CRM element (CRM2) are used to amplify genomic DNA cleaved at a restriction site and ligated to an adapter primer (Shi *et al.* 2010). Transposon display yields marker bands that can be both genetically mapped and sequenced. If the marker sequence provides unique junction sites or polymorphisms, when combined with CENH3 ChIP-seq, the method can be further used to pinpoint functional centromere core domains.

The centromere of the maize B chromosome is an example of a centromere where sequence and marker information could be of great use. The B chromosome is a supernumerary chromosome that has the property of nondisjoining specifically at the second pollen mitosis so that it is preferentially transmitted (Birchler and Han 2009). The entire chromosome is dispensable and has been exploited for multiple purposes including gene mapping, gene dosage analysis, genome engineering, and centromere studies (Nannas and Dawe 2015). The B chromosome contains a unique sequence called ZmBs (*Zea maize* B-specific) or simply the B repeat (Alfenito and Birchler 1993). The B repeat is present in multiple locations on the B chromosome but is most abundant in and around the centromere as assayed by CENH3 staining (Lamb *et al.* 2005; Jin *et al.* 2008). Fine-scale cytological analyses of stretched DNA fibers by fluorescence *in situ* hybridization (fiber-FISH) have revealed a 700-kb CentC and CRM-rich core that is interspersed with blocks of the B repeat (Jin *et al.* 2005).

Some isolates of the B chromosomes undergo frequent centromere misdivision caused by errors in chromosome alignment that result in separation and breakage of the centromere into two parts (Kaszas and Birchler 1996). Most misdivision events result in smaller centromeres as assayed by the quantity of B repeat; however, the size of the centromere also can increase as an outcome of misdivision (Kaszas and Birchler 1998). Transmission of the B chromosome is clearly reduced when centromeres become very small (Phelps-Durr and Birchler 2004), and five of the smaller misdivision-derived centromeres were assayed by fiber-FISH and shown to have deletion breakpoints within the 700-kb B centromere core (Jin *et al.* 2005). Other screens have been used to identify chromosomes where the B centromere has become epigenetically inactivated (Han *et al.* 2006), and then from those lines chromosomes were identified that had regained centromere activity (Han *et al.* 2009). Additional data demonstrate that centromere misdivision can involve the loss and reformation of centromeres at new locations (Liu *et al.* 2015). The molecular underpinning of these events is of great interest; however, there are currently no mapping resources for the B centromere core.

Here we report the first molecular map of the maize B centromere and outline a method for assembling unique sequence from highly repetitive centromere regions. We used CRM2 transposon display (TD) to identify 40 TD markers and mapped them within the centromere using misdivision derivatives. Markers were cloned, sequenced and assembled into a 10.1-kb pseudocontig “minimal map,” which was used to interpret CENH3 ChIP-seq data. We then compared the presence/absence of CENH3-enriched markers in misdivision lines to identify a set of markers that are unique to the functional centromere core. We also generated seven simple polymerase chain reaction (PCR) primer pairs that can be used to score the major domains of the centromere without employing CRM-TD. This important reference information can be used as a basis for interpreting the large collection of known B centromere variants and derivatives. In addition, the B centromere minimal map can serve as a guide for a future full assembly of the B centromere.

## Materials and Methods

### Transposon display

To develop markers on the B chromosome, a modified amplified fragment-length polymorphism approach was used to amplify centromere repeat with the use of CRM2 LTR primers as described previously (Casa *et al.* 2000; Shi *et al.* 2010). Genomic DNA was digested with *BfaI*. For the primary amplification step we used primer CRM2_R1 (5′- GAGGTGGTGTATCGGTTGCT) and *BfaI* + 0 (5′- GACGATGAGTCCTGAGTAG). For selective amplification we used P33- or FAM (fluorescein amidite)-labeled CRM2_R2 (5′- CTACAGCCTTCCAAAGACGC) and *BfaI* + 3 selective bases (5′-GACGATGAGTCCTGAGTAG + NNN). All 64 combinations of NNN were used as an attempt to identify all CRM2 elements in the B centromere. Many of the markers (all of those processed for sequencing) were analyzed on 6% polyacrylamide gels, blotted, and exposed to film (Shi *et al.* 2010). We also used FAM-labeled primers in the same manner as described (Shi *et al.* 2010) by separating the bands using a capillary sequencer and interpreting the results using GeneMarker software. Both methods were used to compare a B73 line carrying the B chromosome with a B73 line without the B chromosome. After identifying B centromere-specific bands in this controlled background, we scored them in (nonisogenic) stocks containing TB-9Sb, MiniB (#9), pseudoisochromosome (PI), Telo2-1(-), Iso3(-), Telo2-2(-).

### Sequencing of TD markers

DNA from B-specific TD bands were extracted from polyacrylamide gels, amplified with preamplification primers, and confirmed using 2.0% agarose gels. Only samples that showed a single band were pursued further. PCR products were purified with a QIAGEN gel purification kit and were either directly sequenced or first cloned into a TOPO TA vector (Invitrogen, Carlsbad, CA) and Sanger sequenced. Two primers were used for the sequencing, a forward primer homologous to CRM2 and a reverse primer for the ligated adapter, resulting in two separate sequences for each marker. Sequence alignment and quality was analyzed with the use of Geneious software (Kearse *et al.* 2012). Although 250 bands were sequenced, a large number were duplicates, and many aligned to the B73 reference genome; these were removed to leave a total of 61 unique markers. As described in the text, 21 of these were subsequently shown to lie outside of the centromere and were not studied further. The remaining 40 sequences described in Table 1 are available in GenBank (KT724890-KT724929).

**Table 1:** CRM2-TD markers mapped to misdivision derivatives

CRM2 TD Markers	TD	TB-9Sb	PI	Telo 2-1(-)	Iso3(-)	Telo 2-2(-)
CRM2-ACC-CCA-AGA-ATA-196	TD1	+	−	−	−	−
CRM2-CCA-375	TD2	+	−	−	−	−
CRM2-CCC-280	TD3	+	−	−	−	−
CRM2-CCC-TCC-342	TD4	+	−	−	−	−
CRM2-GGA-GAC-405	TD5	+	−	−	−	−
CRM2-TAT-GAT-CTT-173	TD6	+	+	−	−	−
CRM2-ATA-CCA-342*	**TD7**	+	+	+	−	−
CRM2-TCC-310*	TD8	+	+	+	+	−
**CRM2-TCT-209**	**TD9**	+	+	+	+	−
CRM2-AGA-214*	**TD10**	+	+	+	+	+
CRM2-CTA-CTG-GTA-247	TD11	+	+	+	+	+
CRM2-CTG-389	TD12	+	+	+	+	+
CRM2-GAT-228	TD13	+	+	+	+	+
CRM2-GTC-188	TD14	+	+	+	+	+
CRM2-CGG-326*	**TD15**	+	+	+	+	+
CRM2-AGC-GGC-257	TD16	+	+	+	+	+
CRM2-AGC-GGC-GAC-382*	TD17	+	+	+	+	+
**CRM2-CTT-256**	**TD18**	+	+	+	+	+
CRM2-TAT-161	TD19	+	+	+	+	+
CRM2-TCC-345	TD20	+	+	+	+	+
CRM2-ACC-ATG-CTG-351*	**TD21**	+	+	+	+	+
CRM2-AGA-138	TD22	+	+	+	+	+
CRM2-AGA-AGG-GGA-TCG-207	TD23	+	+	+	+	+
CRM2-ATC-TCA-220	TD24	+	+	+	+	+
CRM2-CAG-335	TD25	+	+	+	+	+
CRM2-CAT-AGT-227	TD26	+	+	+	+	+
CRM2-CGA-361	TD27	+	+	+	+	+
CRM2-CTC-199	TD28	+	+	+	+	+
**CRM2-GCC-TTC-CCC-TAC-CCT-309**	**TD29**	+	+	+	+	+
CRM2-GGG-186	TD30	+	+	+	+	+
CRM2-GGG-298	TD31	+	+	+	+	+
CRM2-TAC-140	TD32	+	+	+	+	+
CRM2-TAT-CAT-159	TD33	+	+	+	+	+
CRM2-TCC-350	TD34	+	+	+	+	+
CRM2-AAT-GAT-208	TD35	+	+	+	+	+
CRM2-AGC-CAG-CCG-275	TD36	+	+	+	+	+
CRM2-ATG-298	TD37	+	+	+	+	+
CRM2-GAC-AGC-AGT-CGT-ATT-AAT-185	TD38	+	+	+	+	+
CRM2-GTC-247	TD39	+	+	+	+	+
CRM2-AGG-328*	TD40	+	+	+	+	+

Marker names include all the selective base pairs that amplified the sequence. TD indicates the pseudonym for each marker. Presence or absence of the band is noted by a “+” or “−“. Markers noted by an asterisk also were converted to simple polymerase chain reaction markers, and those in bold interact with CENH3 by ChIP-seq.

### Chromatin-immunoprecipitation

For the TB-9Sb lines, we used a modified ChIP protocol developed by Zidian Xie of the Gernot Presting laboratory for 2-wk-old maize seedlings based on a published protocol (Li *et al.* 2012) using antibodies to maize CENH3 (Zhang *et al.* 2013). Briefly, the nuclei were isolated and chromatin digested with 12.5 U of MNase (Affymetrix, 70196Y) and enriched for mononucleosomes. For 9Bic-1 lines, we used the outer tissue of immature ears and different CENH3 antibodies (Zhong *et al.* 2002). Both the TB-9Sb and 9Bic-1 stocks had been backcrossed to the B73 reference line at least five times. We performed CENH3-ChIP on three TB-9Sb plants and one 9Bic-1 plant, with two technical replicates for the 9Bic-1 sample. As controls for false-positive enrichment, we used published B73 CENH3 ChIP and fragmented genomic sequences (Gent *et al.* 2014).

### Illumina library prep and sequencing

For TB-9Sb ChIP samples, libraries for Illumina sequencing were prepared by the University of Missouri Core facility using a NEBNext Ultra Library Prep Kit (NEB, E7370L) and sequenced on an Illumina HiSeq2500 using paired-end sequencing at average 100 nt lengths. The 9Bic-1 sequence libraries were prepared without a kit, but with TruSeq indexes and sequenced at Georgia Genomics Facility, with NextSeq500 High output flow, single-end sequencing at average 150 nt lengths. TB-9Sb and 9Bic-1 sequence can be found at GenBank with the accession numbers SRX1166240 and SRP062947 respectively.

### Sequence alignment and analysis protocol

Reads were processed by removing adapters and trimming for quality, keeping only ≥125 nt for 9Bic-1 and ≥50 nt for TB-9Sb the library. Reads were then aligned to the maize genome plus the B centromere minimal map. The B centromere minimal map was created by joining the sequences of the TD markers in the order shown in Table 1, with 200 Ns separating each marker sequence. Alignment was carried out with Burrows-Wheeler Aligner (Li and Durbin 2009) with MEM alignment option at default except that seed alignment length (-k) was increased to 40 bp. The output of Burrows-Wheeler Aligner-MEM was filtered with SAMtools (Li *et al.* 2009), set at view –bS –q 30 to filter out MAPQ scores lower than 30. The Model-based Analysis of ChIP-Seq 2 software (MACS2) (Feng *et al.* 2012) was used to calculate CENH3 enrichment relative to the genome average using the following parameters; macs2 callpeak -t -f BAM -g hs–outdir -B–SPMR–call-summits. The MACS2 pileup output files were converted to bigwig files (Kent *et al.* 2010) and displayed as fold-enrichment using the Integrated Genomics Viewer (Robinson *et al.* 2011).

To identify core centromere markers in the B minimal map, we compared seven separate sequencing libraries. The B73 fragmented genome and B73 CENH3 ChIP sample (Gent *et al.* 2014) provided a negative and positive control, respectively. The 9Bic-1 line contains an inactivated B centromere (Han *et al.* 2006) and was used as an additional negative control. TB-9Sb carries a complete and active B centromere and was compared to all other lines for enrichment of CENH3 on the B minimal map. For the 9Bic-1 line, we sequenced two technical replicates of ChIP experiment. For TB-9Sb, we sequenced three separate ChIP experiments. Markers scored as interacting with CENH3 showed enrichment for all replicates of TB-9Sb, no more than twofold enrichment for 9Bic-1, but no enrichment for B73 CENH3 or fragmented B73 lines.

### Data availability

The sequence data described here can be found in GenBank (KT724890–KT724929) and the NCBI Sequence Read Archive (SRX1166240 and SRP062947).

## Results

### Identification of B centromere sequences by CRM2-TD

**Figure 1:**
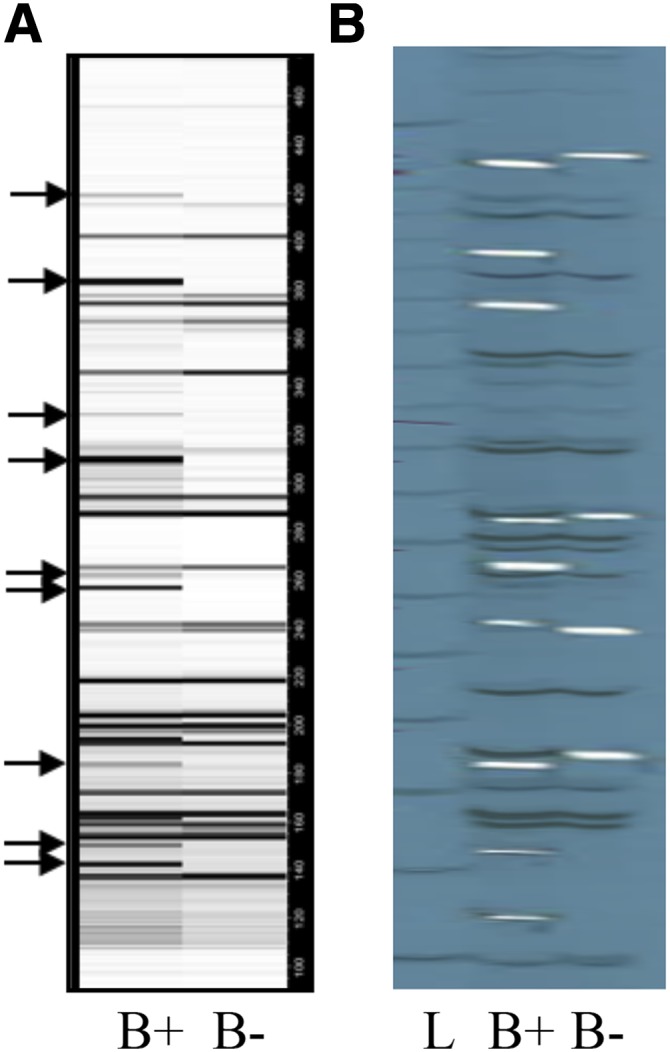
Radio- and fluorescence-labeled transposon display (TD) of CRM2. These data were acquired with the selective bases AGC on the adapter primer. Lanes are labeled with B+ (carrying multiple copies of the B chromosome), B− (control line without B chromosomes), and L (ladder). (A) Fluorescence-labeled TD. Digital data were converted to a pseudogel format with GeneMarker software. Arrows indicate bands that were chosen sequencing. (B) The same adapter primers used in A were 33P-labeled for TD, and the results separated by polyacrylamide gel eletrophoresis and exposed to film. The film was placed back onto the gel, and a blade used to cut through the film and gel; the gel slice was then used for reamplification polymerase chain reaction.

When transposons insert into other repetitive sequences, unique junction sites are created (Luce *et al.* 2006; You *et al.* 2010). We targeted the regions flanking CRM2 using TD, a modified amplified fragment-length polymorphism approach that takes advantage of restriction site polymorphism flanking the conserved LTR sequences of the transposon (Shi *et al.* 2010). CRM2 primers, labeled with radioactive or fluorochrome-labeled nucleotides, were paired with adapter primers that bind to *BfaI* cleavage sites. An additional PCR step using adapter primers with three selective bases was used to reduce the number of bands and improve resolution. To identify bands specific to the B chromosome, we compared the banding pattern from B73, the sequenced reference inbred to a B73 line containing several B chromosomes (called the B+ line, Figure 1).

### Sequence of the TD markers

Each marker unique to the B+ line was extracted from a polyacrylamide gel, PCR amplified, and Sanger sequenced (Figure 1). A total of 250 markers were analyzed initially in this manner. However, after sequencing, we discovered that the selective bases on adapter primers were only weakly selective and many of the bands were duplicates. We ultimately chose a set of 61 markers that are unique to the B chromosome. This collection of markers was then analyzed in a line containing a “miniB” chromosome that contains only the centromere and flanking pericentromeric regions (Kato *et al.* 2005; Han *et al.* 2007). Twenty-one markers proved to be absent on the miniB chromosome, thereby narrowing the number of potential centromeric markers to 40.

All markers contained ∼112 bp of the CRM2 LTR amplified during TD, as well as the adjoining flanking sequence. Nine of the markers contained either the B repeat or CentC sequence as expected for the B centromere core region (Jin *et al.* 2005). Retroelements were otherwise the most common flanking sequences, including CRM1 (eight), Cinful-Zeon (four), CRM2 (seven), Xilon-Diguus (four), Sela (two), CRM3 (two), Doke (two), Huck (two), and one instance each of CRM4, Flip, Gyma, Puck, and Ji (Supporting Information, Table S1). We also observed one instance of a repeating motif (TTTAGGG) that is observed in the B repeat (Alfenito and Birchler 1993).

### Grouping markers within the B centromere using early misdivision derivatives

To make a genetic map of the B centromere, we analyzed a collection of lines with fragmented centromeres produced after centromere misdivision (Kaszas and Birchler 1996, Birchler 1998). All misdivision derivatives were derived originally from a translocation chromosome called TB-9Sb that involves the B chromosome and the short arm of chromosome 9. A derivative of TB-9Sb called PI (for Pseudoisochromosome) was recovered previously (Carlson 1970). This chromosome contains two arms of chromosome 9 centered on a B centromere; however, one arm contains a small knob that was derived from the B chromosome and the other arm does not, making it asymmetrical (Carlson and Chou 1981). Many centromere misdivision derivatives of the PI chromosome were identified on the basis of the behavior of kernel color markers on chromosome 9S (Kaszas and Birchler 1996).

**Figure 2:**
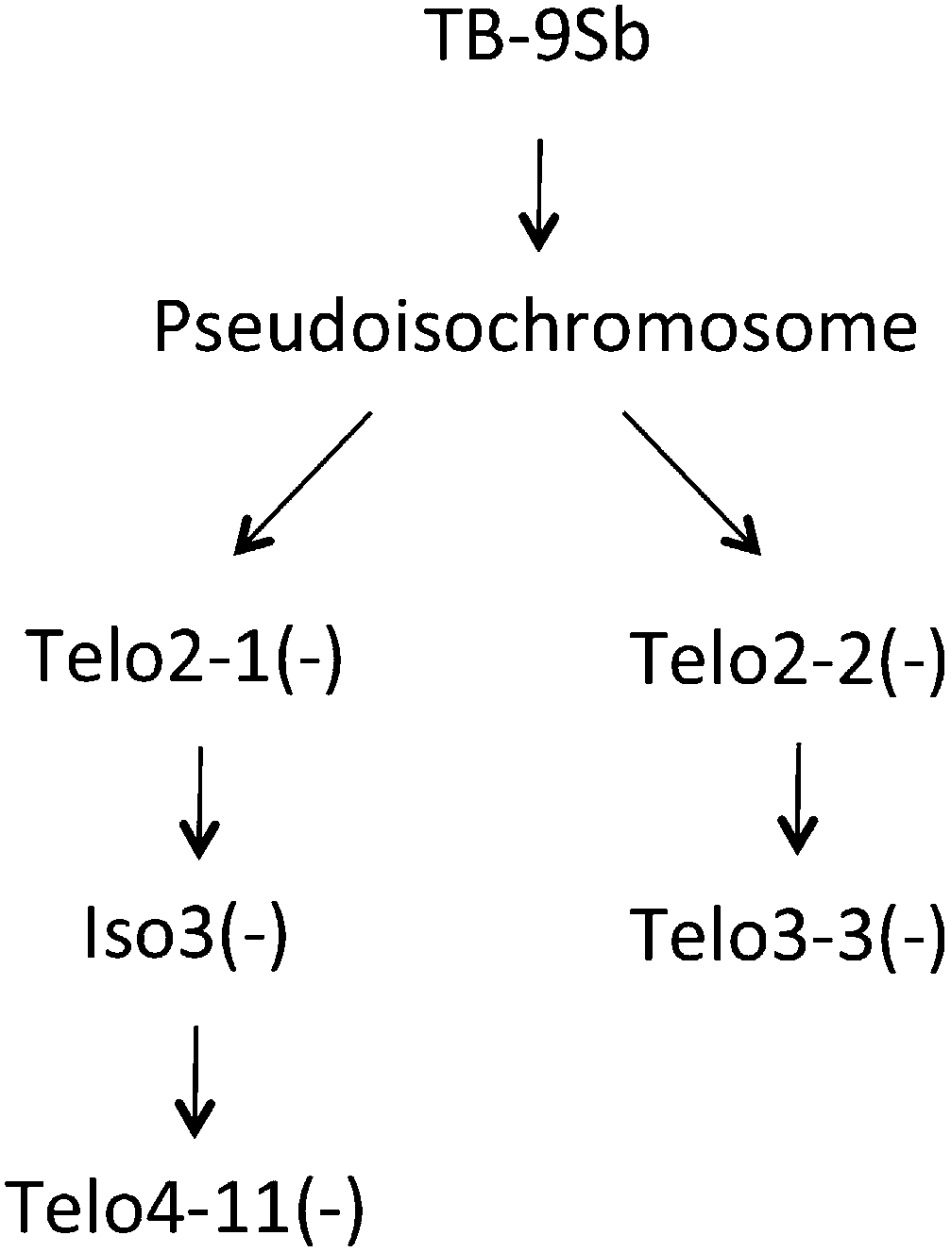
Pedigree of the misdivision lines used. All derivatives were derived from TB-9Sb, which gave rise to the pseudoisochromosome (PI). Two second-generation derivatives [(Telo2-1(-) and Telo2-2(-)] were derived from PI. We also studied two third-generation derivatives [(Iso3(-) and Telo3-3(-)] and one fourth-generation derivative [Telo4-11(-)].

Misdivision derivatives recovered from the PI chromosome are categorized as telocentric “Telo” or true isochromosomes “Iso” with identical arms (ring chromosomes also were recovered but are not studied here). In addition, the chromosomes are differentiated by the presence (+) or absence (−) of the small knob from the PI chromosome. We carried out CRM2-TD on the TB-9Sb progenitor and four early descendants: PI, Telo 2-1(-), Iso3(-), and Telo2-2(-) (Figure 2). The observations are reported in Table 1. We found that five markers differentiate TB-9Sb from PI, one marker differentiated PI and Telo2-1(-), one marker differentiated Telo2-1(-) and Iso3(-), and two markers differentiated Iso3(-) and Telo2-2(-), leaving 31 markers that are present on all five chromosomes. These markers are likely to lie within or close to the 700-kb domain described by Jin *et al.* (2005), but their relative arrangement and orientation within the core domain is not known.

**Figure 3:**
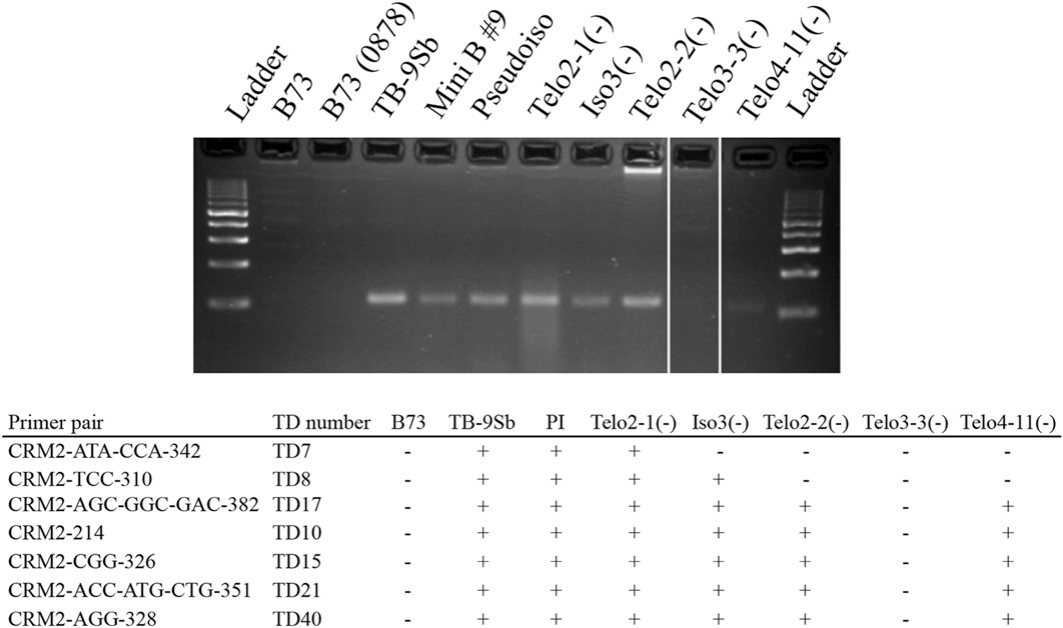
Polymerase chain reaction (PCR) confirmation of transposon display (TD) marker patterns. Upper image shows the amplification of CRM2-AGG-328 (TD40) in the lines studied here using unique junction-junction primers. All lanes were from the same gel (regions where lanes were removed are indicated by white lines). Lower table shows the results from amplifying all seven junction-junction PCR primer pairs. Telo3-3(-) and Telo4-11(-) were not assayed by TD and only assayed using junction-junction markers.

To confirm these patterns, unique PCR primer pairs were developed to sequences within seven TD markers (Figure 3, Table S2). These primers identify unique polymorphisms or straddle the boundaries of different repetitive sequences. For instance, in the case of TD8, one primer lies within CRM2 and the second primer straddles the boundary between a fragment of a B repeat and a Sela retroelement. The presence or absence of the PCR bands confirmed the TD results in all cases. We also assayed two later-generation derivatives using the junction-junction primers, Telo4-11(-) and Telo3-3(-) (Kaszas and Birchler 1998), which were assayed in the previous fiber-FISH study (Jin *et al.* 2005). The patterns of amplification indicated that Telo4-11(-) has the same basic makeup as Telo2-2(-), and that Telo3-3(-) is missing all markers in the centromere core (Figure 3), consistent with the previous study (Jin *et al.* 2005).

### Use of ChIP-seq to identify the centromere core on the B centromere minimal map

The screening method used to identify TD markers ensures that the combination of CRM2 insertion and *BfaI* restriction site are unique to the B chromosome. In addition, the internal sequences between the CRM2 and *BfaI* sites, while generally derived from transposable elements or other repeats, frequently have acquired polymorphism that makes them effectively unique over the length of an Illumina sequence read. To build a map that could be used as a reference sequence, we joined sequences from TD bands in the order shown in Table 1, with 200 Ns separating each marker sequence. The total amount of sequence in this pseudocontig (excluding Ns), which we call the B centromere minimal map, is 10,100 bp.

**Figure 4:**
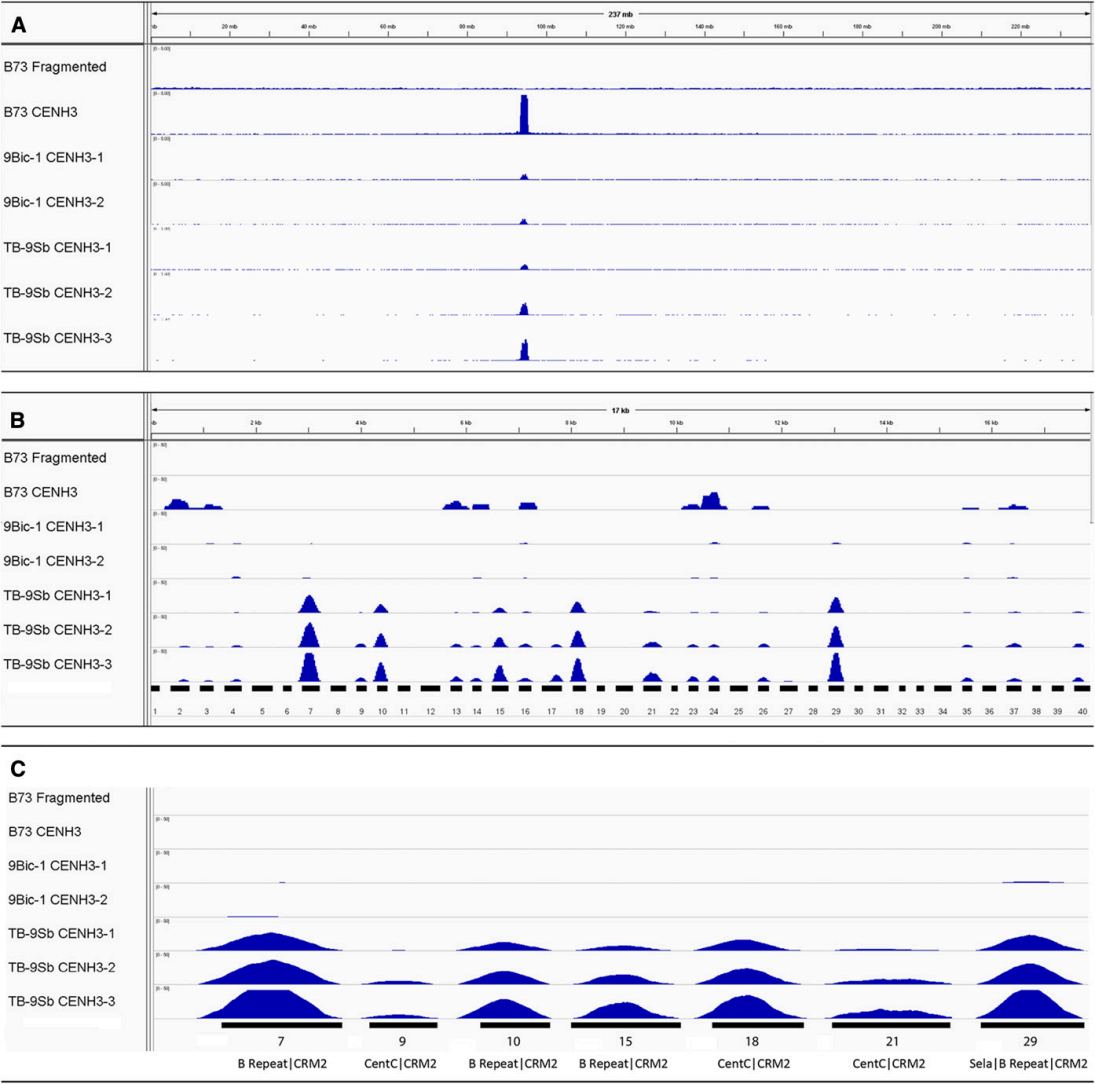
CENH3 ChIP-seq reads aligned to the B73 genome and B centromere minimal map. In all three panels, the first bar shows an alignment to fragmented B73 DNA as a negative control. The second row shows the results of ChIP-seq using B73 tissue, which does not contain B chromosomes. The third and fourth rows show the results from two technical replicates of a plant carrying 9Bic-1, a B chromosome variant with an inactive centromere. The last three rows show ChIP-seq results from three different samples of plants carrying TB-9Sb. (A) Alignment to B73 chromosome 2 showing the centromere position as a single sharp peak. The total length of chromosome 2 is 237 Mb. (B) Alignment to the B centromere minimal map with each marker shown and numbered below. Markers are separated by 200 Ns such that the total length of the sequence shown is 17,900 bp. (C) Enlarged images of the ChIP-seq distributions over the seven markers that interact specifically with CENH3. The repeats within the markers are also indicated.

ChIP was carried out on TB-9Sb seedlings to identify sequences that interact with the centromeric histone variant CENH3. Nuclei from whole seedlings were isolated, MNase-digested, and precipitated with maize CENH3 antibodies (Zhong *et al.* 2002). We also carried out ChIP on two control lines, the B73 reference inbred and a line containing an inactive B centromere called 9Bic-1 (Han *et al.* 2006). Both the TB-9Sb and 9Bic-1 chromosomes were backcrossed to the B73 inbred at least five times. Libraries were created from the precipitated DNA and sequenced via Illumina high-throughput sequencing. The reads were aligned to the B73 genome assembly (version 3) and the B centromere minimal map (Figure 4, B and C and Figure S1). Only unique hits were considered. The levels of CENH3 enrichment were calculated by comparing read depth over centromere markers to the B73 whole-genome average.

Many of the B centromere markers showed significant enrichment for CENH3 in the TB-9Sb sample by a first approximation (Figure 4B and Table S3, enrichment values ranged from 0.1 to 36.6). However, in some cases, alignment also was observed from ChIP-seq samples from B73 or 9Bic-1 control samples, indicating that the internal regions are not unique to the B centromere (Figure 4B and Figure S1) (they are unique relative to the B73 reference genome, but the reference is incomplete in repetitive regions). Other markers were excluded because the ChIP-seq reads did not flank the unique CRM2 insertion point. By these criteria, at least seven markers are unique to the B centromere, show ChIP-seq enrichment only in samples containing an active B centromere, and show uniform ChIP-seq coverage across the junction sites of each marker (Figure 4C and Figure S1). The seven markers are TD7 with 36-fold enrichment, TD9 with threefold enrichment, TD10 with 18-fold enrichment, TD15 with 12-fold-enrichment, TD18, with 23-fold enrichment, TD21 with sixfold enrichment, and TD29 with 31-fold enrichment. Each of the seven markers that interact with CENH3 contain portions of either the CentC or B-repeat (Figure 4C), consistent with the fact that both repeats are abundant within the 700-kb B centromere core domain (Jin *et al.* 2005).

## Discussion

Although the B chromosome is nonessential to maize, it has had a major role in many of the most important studies of plant centromeres (Birchler and Han 2009). Its dispensable nature has made the B centromere amenable to cytogenetic screens that are not possible for normal centromeres, including the selection of deletion events through misdivision and the discovery of entirely inactive centromeres. These studies were facilitated by translocations between the B centromere and the short arm of chromosome 9 (such as TB-9Sb) that made it possible to identify centromere variants by simple visual screens. However, the B chromosome was not included in the maize genome sequence (Schnable *et al.* 2009). Sequences from small portions of the B chromosome have been published (Theuri *et al.* 2005; Cheng 2010), but there have been no systematic studies to identify sequences within the centromere core.

The most detailed study of B centromere structure was a fiber-FISH analysis that identified a putative 700-kb centromere core based on a subset of centromere misdivision events. The centromere core was reported to contain five blocks of B repeat-rich sequence, each flanked by regions of CRM-rich and CentC-rich sequence. The fiber-FISH map has served as a satisfying visual reference and has motivated multiple subsequent studies; however, it provides no sequence information for molecular analysis. Here we used CRM2 TD to capture portions of the B centromere, and sequenced and mapped the markers relative to misdivision derivatives with various centromere sizes. The mapped and concatenated markers were then used to interpret CENH3 ChIP-seq results.

Two forms of data suggest that the B centromere minimal map traverses the functional centromere core. The first form of evidence comes from CENH3 ChIP-seq, which demonstrates that markers of the minimal map interact with CENH3 and therefore identify the functional core (Figure 4). Although a superficial analysis suggests that as many as half the markers may interact with CENH3, for many of these we also observed apparent ChIP in the B73 and 9Bic-1 samples, suggesting that the internal sequences of the markers were not unique to the B centromere. Nevertheless, an interpretation using the strict criteria that no ChIP was observed for the B73 samples, no ChIP was observed for either of the 9Bic-1 replicates, but that ChIP-seq reads covering the entire marker sequence were observed for all three TB-9Sb replicates indicates that at least seven markers lie within the functional B centromere core (Figure 4C).

The second form of evidence that the minimal map identifies the functional B centromere is a general concordance with the fiber-FISH data of Jin *et al.* (2005). By definition, centromere misdivision derivatives must involve a breakage within the centromere core, as the mechanism requires that a single centromere erroneously divides into two parts that separate to opposing poles (Yu and Dawe 2000). Fiber-FISH analysis of derivative Telo2-2(-) revealed a breakage that removed roughly one third of the 700 kb CentC-rich putative core domain (Jin *et al.* 2005). We show here that the formation of Telo2-2(-) involved the removal of nine of the 40 TD markers, including TD9, which interacts with CENH3 (Table 1). Further, according to Jin and coworkers, Telo4-11(-) has a similar structure to Telo2-2(-) and we confirm that here using our set of seven junction-junction markers (Figure 3).

Some early conclusions about the functional importance of the markers can also be made from our data. We can infer that the left-most markers are unlikely to be critical for centromere function, specifically the first seven markers that were deleted in the PI and Iso3(-) derivatives as a result of misdivision (Table 1). Marker TD9 interacts with CENH3 and is presumably part of the B centromere core. Loss of this marker (and presumably other linked sequences) in Telo2-2(-) results in a reduction in transmission of the chromosome to 43% [from the 53% observed for Iso3(-)]. Derivatives that show lower transmission than 43% are rare and tend to be unstable (Kaszas and Birchler 1998). Telo3-3(-) is the best studied of the small, unstable category of misdivision derivative (Jin *et al.* 2005). Importantly, we show here that Telo3-3(-) lacks the entire core domain as assayed by specific PCR markers. Recent data corroborate this observation (Liu *et al.* 2015), showing that there are no detectable CRM elements in the Telo3-3(-) derivative. Although a few small arrays of the B repeat are still present in Telo3-3(-) (Kaszas and Birchler 1998) and were originally thought to be responsible for the low transmission of this chromosome (Jin *et al.* 2005), Liu *et al.* (2015) have shown that the Telo3-3(-) chromosome has acquired a neocentromere in noncentromeric regions close to the original the B centromere. Taken together, the results indicate a core domain identified by markers TD8 through TD40 of the B centromere minimal map is required for centromere function, and loss of this domain leads to centromere loss and the formation of new centromeres.

The availability of simple PCR markers (Figure 3 and Table S2) and 10.1 kb of sequence data derived from the B centromere core will now make it possible to interpret other small unstable derivatives, and ultimately develop a full physical map of the B centromere for additional studies of centromere structure and function. Such short sequence tags, when combined with sequences from a B chromosome-containing BAC library, or newer single-molecule sequencing technologies (Pendleton *et al.* 2015), should make it possible to rapidly identify the functional centromere core within long, otherwise un-anchored contigs. We anticipate that a complete B centromere assembly will allow us to more accurately interpret the complex events underlying centromere misdivision (Kaszas and Birchler 1998) and inactivation (Han *et al.* 2009), as well as more accurately deploy engineered B chromosomes as gene delivery platforms (Birchler 2015).

## 

## Supplementary Material

Supporting Information
