# Supplementary material for: Generation of a Maize B Centromere Minimal Map Containing the Central Core Domain
Source: G3 (Bethesda). 2015 Oct 26;5(12):2857–64. doi: 10.1534/g3.115.022889 (PMC4683656; doi:10.1534/g3.115.022889)
Supplement: Supporting Information [file supp_5_12_2857__index.html]

Generation of a Maize B Centromere Minimal Map Containing the Central Core Domain — Supporting Information 

# Generation of a Maize B Centromere Minimal Map Containing the Central Core Domain

## Supporting Information for Ellis *et al.*, 2015

**Files in this Data Supplement:**

- Figure S1 - CENH3 fold-enrichment over TD markers in B73, 9Bic-1, and TB-9Sb. (.pdf, 55 KB)
- Table S1 - Sequence homology of the TD markers. (.pdf, 16 KB)
- Table S2 - B centromere-specific junction-junction primer pairs. (.pdf, 13 KB)
- Table S3 - MACS2 fold-enrichment for each TD marker on the minimal map. (.pdf, 20 KB)
