# Supplementary figures and images for: Generation of a Maize B Centromere Minimal Map Containing the Central Core Domain
Source: G3 (Bethesda). 2015 Oct 26;5(12):2857–64. doi: 10.1534/g3.115.022889 (PMC4683656; doi:10.1534/g3.115.022889)

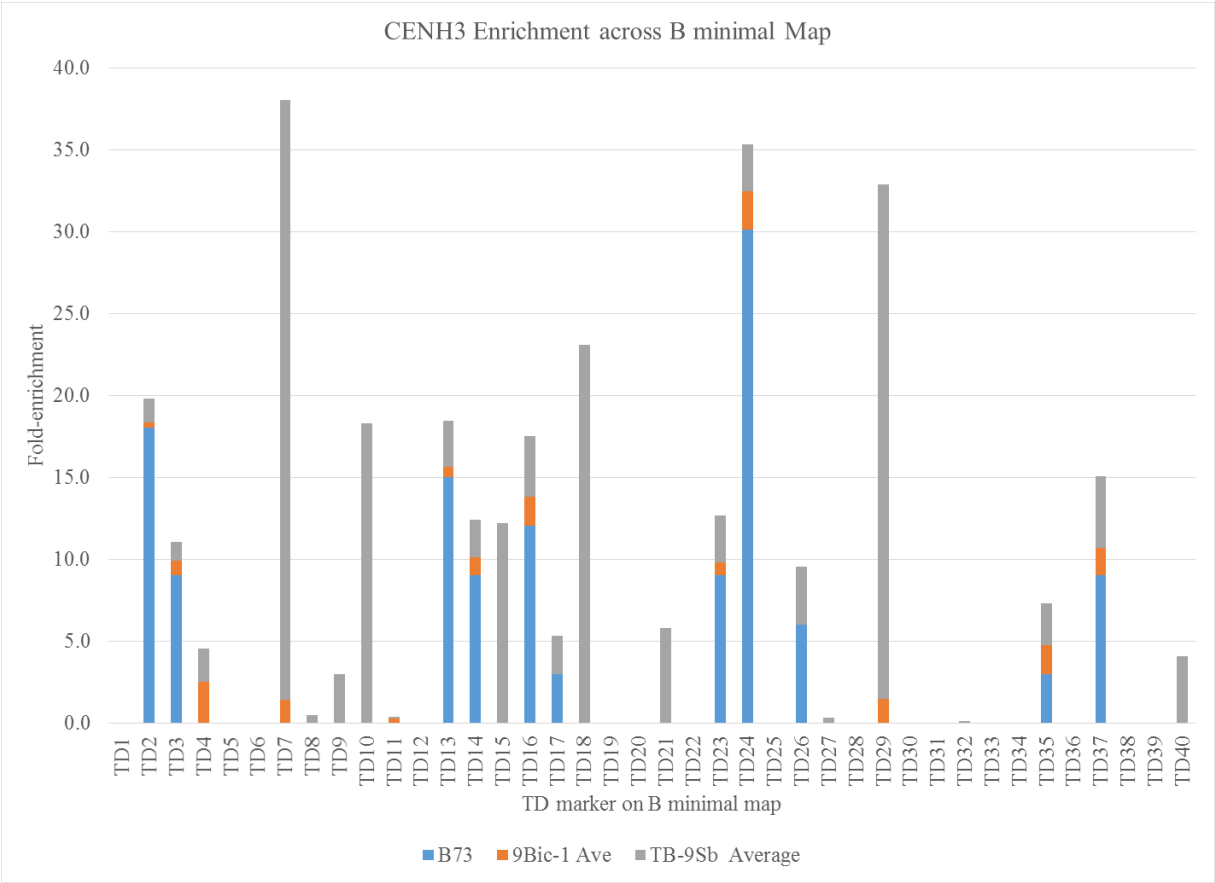

**Figure S1:** CENH3 fold-enrichment over TD markers in B73, 9Bic-1, and TB-9Sb.

Supplement: Supporting Information [file supp_g3.115.022889_FigureS1.pdf]
