# Supplementary material for: Generation of a Maize B Centromere Minimal Map Containing the Central Core Domain
Source: G3 (Bethesda). 2015 Oct 26;5(12):2857–64. doi: 10.1534/g3.115.022889 (PMC4683656; doi:10.1534/g3.115.022889)
Supplement: Supporting Information [file supp_g3.115.022889_TableS1.pdf]

Table S1. Sequence homology of the TD markers. Identity of the CRM2-flanking sequence was interpreted using Blastn. All markers contain sequences homologous to known retroelements. Some markers also show homology to other known centromere repeats.

Marker	Retroelement homology	Other Repeats
TD1	Huck	
TD2	CRM1	
TD3	CRM1 , CRM4	
TD4	Huck	
TD5	Xilon-Diguus	
TD6	CRM1	
TD7	CRM2	B repeat
TD8	Sela	B repeat
TD9	CRM2	CentC
TD10	TTTAGGG	B repeat
TD11	Ji	
TD12	CRM1	
TD13	Xilon-Diguus	
TD14	CRM3	
TD15	CRM2	B repeat
TD16	CRM1	
TD17	CRM1	
TD18	CRM2	CentC
TD19	CRM2	
TD20	Flip	
TD21	CRM2	CentC
TD23	CRM1	
TD24	Xilon-Diguus	
TD25	Doke	
TD26	Puck	
TD27	Doke	
TD28	Xilon-Diguus	
TD29	Sela	B repeat
TD30	CRM1	
TD31	Gyma	
TD32	No repeats found	
TD33	No repeats found	
TD34	No repeats found	
TD35	CRM3	
TD36	Cinful-Zeon	
TD37	Cinful-Zeon	
TD38	Cinful-Zeon	
TD39	Cinful-Zeon	
TD40	CRM2	CentC
