# Supplementary material for: Generation of a Maize B Centromere Minimal Map Containing the Central Core Domain
Source: G3 (Bethesda). 2015 Oct 26;5(12):2857–64. doi: 10.1534/g3.115.022889 (PMC4683656; doi:10.1534/g3.115.022889)
Supplement: Supporting Information [file supp_g3.115.022889_TableS2.pdf]

Table S2. B centromere-specific junction-junction primer pairs.

TD marker	TD Name	Primer Name	Sequence
CRM2-ATA-CCA-342	TD7	CRM2-ATA-CCA-342-6-F	AAACGCTATAGGACAGGCCC
		CRM2-ATA-CCA-342-6-R	TCTTTGGAGGCTGTAGTCGG
CRM2-TCC-310	TD8	CRM2-TCC310-5-F	CATAAACCCTAAAGCCCAAACC
		CRM2-TCC310-5-R	TCTTTGGAAGGCTGTAGTCGG
CRM2-214	TD10	CRM2-AGA-214-5-F	CGGGTGCACATCAACTAACC
		CRM2-AGA-214-5-R	GAGTTTGGGTTTTTGGATTTATGG
CRM2-CGG-326	TD15	CRM2-CGG-326-4-F	GGGTGCACATCAAGAACCAT
		CRM2-CGG-326-4-R	CGAAAACACCCCAAAGATGA
CRM2-AGC-GGC-GAC-382	TD17	CRM2-AGC-GGC-GAC-382-1-F	CCAACGGGTGCACATCAC
		CRM2-AGC-GGC-GAC-382-1-R	CCCCCTGCTGTTGTTAACCT
CRM2-ACC-ATG-CTG-351	TD21	CRM2-ACC-ATG-CTG-351-6 F1	CTAGTCGATTCGGCATGTTCGTTGCG
		CRM2-ACC-ATG-CTG-351-6 R2	GGTGCACATCATTTCGCGCAATTCAG
CRM2-AGG-328	TD40	CRM2-AGG-328-4 F3	CGGTAACGTACGGCAACG
		CRM2-AGG-328-4 R2	CATCAAGAACCATTTCTACGTTTATCG
