# Supplementary material for: Generation of a Maize B Centromere Minimal Map Containing the Central Core Domain
Source: G3 (Bethesda). 2015 Oct 26;5(12):2857–64. doi: 10.1534/g3.115.022889 (PMC4683656; doi:10.1534/g3.115.022889)
Supplement: Supporting Information [file supp_g3.115.022889_TableS3.pdf]

Table S3: MACS2 fold-enrichment for each TD marker on the minimal map.

TD Marker	B73	9Bic-1 Average	TB-9Sb Average
TD1	0.0	0.0	0.0
TD2	18.1	0.3	1.4
TD3	9.0	0.9	1.2
TD4	0.0	2.6	2.0
TD5	0.0	0.0	0.1
TD6	0.0	0.0	0.0
TD7	0.0	1.4	36.6
TD8	0.0	0.0	0.5
TD9	0.0	0.0	3.0
TD10	0.0	0.0	18.3
TD11	0.0	0.3	0.1
TD12	0.0	0.0	0.0
TD13	15.1	0.6	2.8
TD14	9.0	1.1	2.3
TD15	0.0	0.0	12.2
TD16	12.0	1.8	3.7
TD17	3.0	0.0	2.3
TD18	0.0	0.0	23.1
TD19	0.0	0.0	0.0
TD20	0.0	0.0	0.0
TD21	0.0	0.0	5.8
TD22	0.0	0.0	0.0
TD23	9.0	0.8	2.9
TD24	30.1	2.4	2.9
TD25	0.0	0.0	0.1
TD26	6.0	0.0	3.5
TD27	0.0	0.0	0.4
TD28	0.0	0.0	0.0
TD29	0.0	1.5	31.4
TD30	0.0	0.0	0.0
TD31	0.0	0.0	0.0
TD32	0.0	0.0	0.1
TD33	0.0	0.0	0.0
TD34	0.0	0.0	0.0
TD35	3.0	1.8	2.6
TD36	0.0	0.0	0.0
TD37	9.0	1.7	4.4
TD38	0.0	0.0	0.0
TD39	0.0	0.0	0.0
TD40	0.0	0.0	4.1
